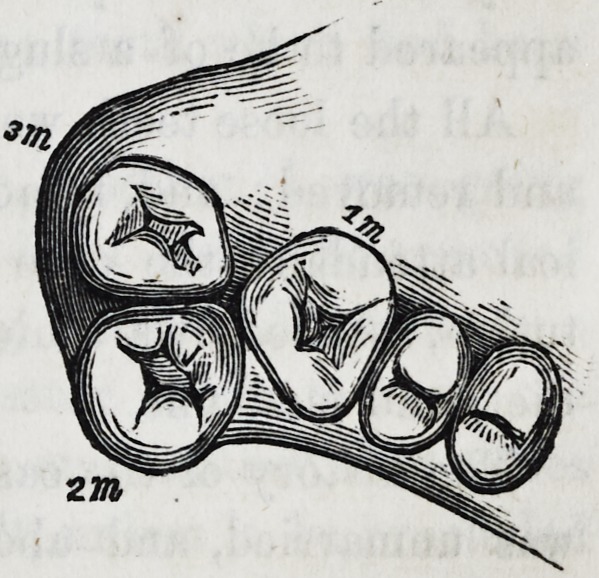# On Irregular Teeth

**Published:** 1853-10

**Authors:** C. Spence Bate


### ARTICLE XV.

*On Irregular Teeth.* By C. SPENCE BATE, ESQ.

REGULARITY of the teeth has only been considered of importance worthy of special interference where it exists within reach of the eye of others, being wholly unworthy of attention if situated further back in the mouth than may be exposed by a laugh.

As far as it concerns the appearance only, no doubt this may be quite correct; but the same injurious result to the teeth must take place in an aggravated form far back in the mouth, from a crowded state of the teeth, as we perceive to exist with those in front.

But since it is a rule that, when any one of the anterior teeth is placed irregularly in the jaw, we should restore it to its normal position at the expense of one or more of the posterior, it must, of course, follow, that when these latter are the subjects of irregularity, they should themselves be removed, unless it should be found that the next to them are carious, when it should be a matter of consideration for the dentist consulted to judge how far desirable interference might be of advantage.

The teeth posterior to the canine, which are at all liable to be so affected, are the bicuspids and the second and third molars. I do not recollect an instance of irregularity of the first molar, which tooth, being itself developed from the primary dental groove, approximates the deciduous set in character, among which history has not yet furnished us with any cases of decided irregularity.

Beyond the tendency to caries which an undue pressure of the teeth upon one another induces, there are other evils of a secondary character which are capable of being produced; for instance, a Mr. T——, who has been known to me for many years, has a second under molar, with the crown projecting into the mouth, so that the facial wall of the tooth becomes the grinding surface in ordinary mastication.

This gentleman is of a strumous constitution; and it almost invariably results, that, upon any debility of the system, the surface of the tongue brought into contact with the irregular tooth, excoriates, and forms a more or less sluggish ulcer; as yet, no greater injury has been the result; but, when I remember the sad termination of a very parallel case, which is detailed below, must confess that I believe the history of this irregular second molar has yet to be written.

Mrs. G., a lady about 55 years of age, was sent to me by my friend Dr. Nicol, she at the time suffering from a painful affection on the side of the tongue. On examination, I found it to consist of three depressions, the centre being the most important, at the bottom of which ulceration had commenced; these depressions corresponded with three teeth, they fitting each respective hollow in the tongue as if they were originally impressed by them; above these ulcers was a hard, scirrhous-like ridge, traversing the length of the tongue beyond each extremity of the ulcer, and which projected so far as to be constantly caught in the bite, to her great pain and annoyance. Upon turning attention to the teeth, which were quite sound, the second molar was observed to lean considerably into the mouth; but it was not a case of original irregularity of that tooth, but was a distortion caused by the extraction of the first molar many years before. The pressure of the posterior tooth, together with the natural absorption of the alveolar processes from the region of the first molar, had caused the second molar to fall forwards, and to occupy the space of the removed tooth, in which position it had established itself with considerable assimilation, and required some careful observation to establish its identity.

Although the crown of the tooth occupied the position of the first molar, it—as is invariably the case under similar conditions—approximated the bicuspid somewhat more than its own fang, and thus gave to the tooth a cant forwards, and an oblique direction to the crown, causing the tooth to lean in towards the tongue, the anterior corner being most in advance of the rest of the teeth.

In order to relieve the tongue, it was thought expedient to extract the second molar, and, at a later period, all the teeth upon that side of the mouth were removed; but, unfortunately, the disease, which had been progressing for about five years, had gained upon the constitution, ulceration of the tongue progressed rapidly, and death was the result, about nine months after I made my acquaintance with it.

The history of parallel diseases of the tongue, originating in laceration from a fractured or carious tooth, can scarcely be called uncommon; but such a fatal termination, resulting from so trifling a cause as an irregular tooth, I am not aware has been previously recorded.

That such irregularity might, and probably does, exist in the mouth of many with impunity, is of course, to be supposed, and would, in all probability, have been so in this case, had not there been a tendency in the system for such predisposition; but on the other hand, it is but too probable, had not the exciting cause been present in the character of an irregular irritating tooth, the disease might have lain dormant for years, or perhaps altogether.

If we then consider the little sacrifice that it would require, is it not a duty, that, when any irritation is exhibited in the soft parts of the mouth from the presence of an irregular tooth, the tooth should be removed as early as the patient can possibly be induced to part with it?

But it is the third molar, or dens-sapiens of the posterior teeth, which is the most liable to malposition, sometimes growing into the cheek, and inducing considerable ulceration of the soft parts. I have a cast of the upper jaw, in which the dens-sapiens must have been developed in the same alveolus as the second molar, or immediately beneath it, so that, when the former had progressed so as to make its way through the gums, it pushed the second molar out of the way external to the arch, the third molar occupying its place in immediate contact with the first; a case more extraordinary still was published by Mr. Tomes, in the *Medical Gazette*, where the third molar occupied an inverted position in the jaw, immediately below and interlacing its fangs with those of the second molar.

Perhaps of all the evils which result from irregularity in the teeth, none are so painful and serious in their results as that which is possible to ensue from the abnormal position of the third molar, and which, from some cause, probably dependent upon our artificial habits, is far more frequently irregularly planted in the jaw among civilized tribes than otherwise; and often when perfectly in a line with the rest, they impinge so tightly against their neighbor as to be the source of pain, not unfrequently amounting to acute neuralgia.

This I believe to be the true source of a mysterious toothache, liable to be confounded with neuralgia, but which the best writers on dental surgery are either silent about or candidly confess their inability to fathom, owing to the conception of the existence of pain being seated far from the origin.

Having myself been a sufferer, cannot do better than relate my own case; the more so, since it was the first to draw my attention to the origin of what has hitherto been a mystery; the only treatment supposed to be of any use was the loss of a sound and valuable tooth.

For two days and nights was the subject, some ten years since, of a severe gnawing, persistent pain, situated in the first molar tooth, on the left side of the inferior maxilla. The tooth had been filled, but at so early a period that the disease could not have proceeded so far as to affect its internal organization. The remainder of the teeth in the mouth were likewise healthy. Upon making a more thorough examination, the third molar was perceived to be making its way through the gum, producing much irritation in its immediate locality; slightly touching it with a probe, produced severe pain for some time. I requested a medical friend to apply the lancet to the part, which he did freely, the application of which induced considerable pain, which was more consciously felt about the temple and ear than in the immediate locality of the tooth itself.

Next morning, the edges of the incision, made by the lancet, appeared to be sloughing, and the inflammation about the tooth much on the increase. The lancet was again used, and passed quite round the tooth, so as to insure its being freely liberated from the integuments, which, in the region of this tooth, are generally tough and in quantity. Not being content with this, my friend passed the lancet along the gum on each side of the teeth, as far as the first molar, in which, originally, the pain was felt. Likewise, took an aperient draught. Within a short time, the edges of these last incisions exhibited signs of undergoing the process of disorganization. In the evening, leeches were applied to the part affected, and I took a sleeping draught, fearing lest, as the night before, I might be kept awake.

In the application of the leech, I was surprised to find that I was not conscious of perceiving the bite of the animal, and it was not for some minutes, that I was aware, that one had preferred the side of my tongue as a more desirable spot for a repast, than the inflammatory surface round the molar tooth.

The morning after their application, the bites on the gums, but not the tongue, were marked by similar white edges of dead tissue, as appeared along the incisions made by the lancet; also, a considerable way along the internal surface of the cheek, which, being swelled from the extension of the irritation, having been slightly excoriated from the friction induced by its coming in contact with the teeth, was exhibiting unhealthy symptoms. In fact, an inflammatory sloughing ulcer was spreading fast, and looking very angry. The tongue became dry and furred, the breath offensive, the pulse beat feverishly, and I felt generally ill. The aperient was repeated, and used a gargle of potassæ nitras, and seriously entertained thoughts of removing the original exciting cause; but it was considered, both by my friendly attendant and myself, that the removal of the third molar at this period could not avail any thing beyond copious blood-letting; for we felt secure in the opinion, that the present disturbance had had its primary cause removed by the free use of the lancet. (Such was the opinion then.) Another day passed, and no benefit appeared to have been derived from the present treatment. In fact, the wound had spread, passing now down the internal wall of the lower jaw, forward, to the second bicuspid tooth; along the external gum, to an equal extent; along the cheek to the angle of the mouth,—passing along the external gum of the superior jaw, and proceeding, internally, upon the posterior palate.

The local application was now changed, by my friend's desire, for a solution of the chloride of soda, while two tablespoonfuls of aperient mixture were taken every two hours.

Next morning, the good effect of this treatment was perceptible, in the evident arrest of the ulcer, and the freedom from pain, which had almost ceased, within two hours after the use of the present gargle. After a day or two the ulcer assumed a sluggish appearance, but which readily healed under the influence of a solution of alum.

At the time, I attributed the full extent of the disarrangement to the delay in the use of the lancet, owing to the seat of pain directing our attention to a spot, somewhat remote from the cause; but the subsequent history of this case, together with observation upon parallel cases for many years, have convinced me, that the cause of pain was the great pressure upon the teeth, by the protrusion through the gums of this new molar.

I say the subsequent history, insomuch that a return of pain was experienced, with every disarrangement of the general system, for some time after the tooth was fully developed, and was finally removed by the passing of a file between it and the first bicuspid, which was done in order to make certain that caries did not exist in a position invisible to the eye. On the application of the file, perfect relief instantly followed.

I shortly afterwards had a better case, in order to test this important fact in the treatment of the teeth. It was a young lady, whose third molar was protruding through the gum. I first tried every recognized treatment, such as lancing the gum, etc., taking care that no flaps of the incised gums should remain, which might again unite above the tooth; but for a long time no advantage beyond temporary relief was obtained. I then passed a dividing file between it and the second molar, the result was partial relief, long before the operation was complete, and perfect freedom from all pain as soon as the file had completely separated this tooth from the next. About a fortnight afterwards, she returned to me, suffering, perhaps, quite as much as previously, she believing the pain to exist between the second and first molar.

Upon looking into the mouth, I found that every trace of the application of the previous filing was obliterated by the third molar being in perfect contact with the approximal surface of the second.

I again repeated the operation of filing; but, not contenting myself with passing it between the second and third, I also cut down between the first sound molar; since which period, about seven years have passed; I have seen her frequently, and she has complained of neither pain nor ache, all the teeth being free from caries.

But the most self-evident case of the immense power which the third molar possesses, by lateral compression of others in the same jaw, was exhibited in the mouth of a lad, in which, in order to complele its own development, the third had thrust the second molar right out of its line of growth, itself occupying the normal position of the latter, as represented in the cut.

This case was brought under my notice, in consequence of severe pain; the second molar, from its position, being useless, was removed, which afforded instantaneous relief. All the teeth were free from disease.

But the most important case of the kind which has been brought under my observation, was that to which the following history refers:

When Miss B. first called on me, I found the two central and the left lateral teeth so denuded of the gums, from the unhealthy absorption of the alveolar processes, that they were so loose as to be scarcely able to retain their places; the wound which surrounded the loose teeth was connected with an ulcer, near the base of the frenum labii, which, upon probing, I found to be connected by a sinus with another ulcerative opening near the mesial line, a little anterior to the suture which unites the palatal process of the maxillary with the palate-bones, and both with another which debouched into the nasal passage.

At this time the ulcer in the mouth, which, from its position, was the most important, was by no means large, but still admitted of small fragments of necrosed bone to come away through it, as also, not unfrequently, was the case by the one first named, that is, at the base of the superior incisor teeth, and from all of which constantly oozed fetid pus. Round the edges of the wound, the color of the ring which marked the boundary of the inflammatory action, was of a purple hue, more particularly so around the palatal ulcer, which, together with the rest, appeared to be of a sluggish character.

All the loose teeth were looked upon as sources of irritation, and removed; and, in accordance with the opinion of her medical attendant, the cure was left to the strength of her constitution, assisted by a tonic treatment, air, exercise, strengthening diet, and medicine.

The history of the case, as related by the patient, who then was unmarried, and about twenty-two years of age, was, that she suffered from cutting her wisdom tooth on the left side upper, during this period, or shortly after, she became conscious of a swelling in the roof of the mouth, but which gave her neither pain nor inconvenience; consequently, it passed unattended to for some time; but, ultimately, finding that it did not improve, she was induced to consult a druggist, who applied some preparation of iodine* locally, and gave her medicine to take.

It was about twelve months after the commencement of the disease, that it was brought under my notice, from which time, for four or five years, pieces of bone came away at intervals, but, ultimately, the disease appeared to be arrested, leaving, as the result, a communication between the nose and mouth about the size of a fourpenny piece, to the great detriment of her powers of speech, except for the application of artificial assistance.

The ulcer in the front of the mouth had almost, though not quite, healed. When I last saw her, no more teeth becoming involved in the waste of their osseous support, although the left canine is denuded of all support to near the extremity of the fang upon that side which is next the ulcerated portion.

The analogy of this case with others that I have met with in practice, would seem to suggest a similarity of cause; viz. palatal abscess from a carious tooth; but in this case, there was no carious tooth to which the disease could be referred, the wisdom tooth being itself perfect, and in her head up, I believe, to the present time; and since her social position is such as must preclude a doubt to cross the mind of its arising from any illegitimate source, I am induced to believe, that the palatal swelling was produced by the irritation caused by the pressure of the third molar upon those teeth which had been previously developed.

It may be a matter for consideration, whether (I was going to add, in all cases) the third molar had not better be removed from the mouth, the other teeth being perfect; its value as a masticating organ is nil, and its pressure tends to keep the others in such close approximation with one another, that caries in the interstices of many must result, unless it be precluded by surgical assistance, or the early removal of this last developed tooth; particularly, this should be taken into consideration when its preservation entails the application of the file upon

***The topical application by iodine, is conjectural from her description of the material, and from conversation with the druggist employed.**

(as in a case previously mentioned) other than the tooth itself; that is, how far the injury done by the application of the file between the first and second molar was compensated by the cure and exoneration from the pain of extracting the third molar.

I am much inclined to think, that the kindest plan is the early removal of the tooth; yet, I confess, that it is pleasant to our humanity to be able to afford relief, as was the case but a few days previously to this being written. A young girl, about twenty years of age, called upon me in apparently the most excruciating agony: every tooth, upon the closest examination, was found to be typically perfect. The pain to her feelings existed between the second biscuspid, and the first molar on the right side upper. Observing this, I passed a dividing file up between them, carrying a safe side towards the molar; that is, cutting most from that tooth, which, in the history of the teeth, is shown to be least valuable. The operation was described as pleasant, and the cure resulted immediately upon the completion of the perfect separation. No return of pain has taken place.*

I could detail cases of this kind in great numbers, since, for the last eight years, it has been somewhat a favorite plan of treatment; but to do so would be merely a repetition of parallel data with similar results.

I am aware, that it should be a golden rule with the dentist, that a file or instrument that has the power to wound, should not be brought into contact with a healthy tooth; but in cases such as these, severe pain liberates an operator from the trammels of general laws in practice; and the difference between an able practitioner in every department of surgery, and any other, exists in the capability of knowing when general rules are applicable, or when and how they confine the powers to cure.

* It is not only in such cases that I find filing important, but also in carious teeth. Where pain is occasioned by contact with the other jaw, the pain is almost certain of being removed by isolating the diseased tooth from its neighbors.

The surgical experience of those most esteemed in dental practice confesses to the fact, that many a healthy tooth has been removed, the operation being the result of intense pain, and no sign of disease either before or after the removal of the tooth has been at all discoverable. Again, similar cases in which an attempt has been persisted in for some time to retain the tooth, even at the expense of considerable pain, the hope being that resolution of the inflammatory action may take place, and the disease subside of itself; but, unfortunately, the overlooked cause still persisting, the local irritation has passed into a systemic one; and, instead of resolution, suppuration of the irritated pulp of the healthy tooth has resulted from the treatment.

Presuming, upon the experience of many years, that if a severe pain exists in a tooth,—say the biscuspids, molars, incisors, or, as I have more frequently found to be the case, described as being between two,—and, upon careful investigation, no caries can be detected in any of the teeth upon that side of the mouth in either jaw which may lead to the conviction, that the pain experienced was sympathetic from such caries, experience and careful observation have convinced me, that the pain felt originates from an undue pressure of the teeth against each other, the exciting cause being most commonly due to the progressive development of the third molar, not so much at the time of its protrusion through the soft tissues as when it has so far advanced in its development, as to bring the broadest diameter of the crown on a level with that of the teeth situated anteriorily to it. If the third molar be distorted in its position, or is such in growth, that there can be little hopes of its becoming an efficient organ,—the duties of which, if it does not fulfil,—its presence must prove worse than useless, and, therefore, it should be removed. But, as not unfrequently happens, the patient's fears, aided by the consciousness of the distant seat of pain, frustrate the surgeon's judgment, it is then that I would urge its pressure against the next being removed by a file being passed between it and the second molar, and this part of the operation to be performed previously to that of a file being passed between those teeth which occupy the seat of pain; and not unfrequently it will be found to preclude the necessity for the latter being done, and, if so, the most valuable teeth are preserved uninjured.

Another test by which the truthfulness of the existence of abnormal pressure may be diagnosed, is to be found in the increased pain being given, or its reproduction from cessation caused, by the introduction of a thin wedge-shaped instrument between either the teeth affected, or between the second and third molar. This instrument increases the pressure, and, therefore, increases the pain; and the true origin of the disease is palpably manifest.

Generally, the operation of the file in such cases, is described as far from producing so disagreeable a sensation as, under ordinary circumstances, and the completion of the operation is always instant relief. Sometimes, if the enamel be thin, or the approximal surfaces lie very parallel to each other, so that the file would have to cut its whole way to the gums coming into contact with the dentine and peridental membrane at the extremity of the enamel, or from some other cause the tooth be exceedingly sensitive; the operation, which would otherwise be very tedious and painful, may be greatly relieved by the local application of either chloroform, or strongly saturated solution of camphor in rectified spirits of wine.

Of course, here, as in all long operations with the file, the frequent application of cold water, by precluding the file and tooth from becoming hot by friction, renders the operation safer and more pleasant to both the operator and patient.

## Figures and Tables

**Figure f1:**